# The Icy Realm of the Rime

**DOI:** 10.3201/eid1702.AC1702

**Published:** 2011-02

**Authors:** Polyxeni Potter

**Affiliations:** Author affiliation: Centers for Disease Control and Prevention, Atlanta, Georgia, USA

**Keywords:** Art science connection, emerging infectious diseases, art and medicine, The Icy Realm of the Rime, The Polar Sea, Caspar David Friedrich, influenza, Arctic, about the cover

**Figure:**
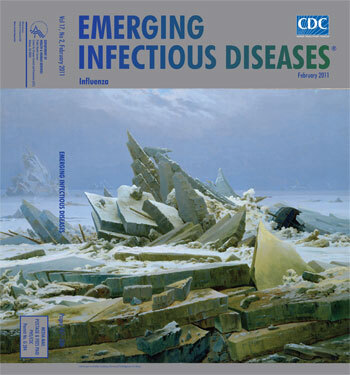
**Caspar David Friedrich (1774–1840) *The Polar Sea* (1824) Oil on canvas (97.8 cm × 128.3 cm)** Hamburger Kunsthalle, Hamburg, Germany/The Bridgeman Art Library

The “taciturn man from the North” is how his contemporaries described Caspar David Friedrich, referring to his melancholy, or in his own words, his “dreadful weariness,” especially in later years. Loneliness pervaded his work as well as his life, which was marred by early deaths in the family―of his mother when he was seven and several siblings, among them, a young brother, who drowned in a frozen lake, according to some, trying to rescue him.

Friedrich was born in Greifswald, then Swedish Pomerania, on the Baltic coast of Germany, the son of a candle maker and soap boiler in a family of 10 children. As a youth he studied with architect and painter Johann Gottfried Quistorp but later moved to Copenhagen to attend the Academy, one of the leading centers of art in Europe, and eventually settled in Dresden. His training in the neoclassical tradition relied on extensive preliminary studies, drawings, and sketches to depict the physical world and is reflected in the disciplined quality of all his works. But while his landscapes were always actual studies of nature, they were more than a representation of nature.

This man, who according to his contemporaries discovered “the tragedy of landscape” and gained by it fame in his own time, soon embraced an untested individual approach to painting, despite a lingering attachment to the systematic techniques of his training. “The artist should paint not only what he sees before him, but also what he sees within him,” he wrote. This belief was rooted in his view of nature as a subject itself worthy of study, imbued with spiritual qualities and portrayed entirely without human presence, not as backdrop but as protagonist. His interest was not in the beauty of nature alone but in what the romantics called the sublime―powerful natural phenomena: snowstorms, impenetrable fog, impassable mountains―generating conflicted feelings of wonder and helplessness, which he could sense and capture with symbols and allegorical elements.

Viewing and presenting the landscape in an entirely new way was Friedrich’s main innovation. He turned the mountains, forests, and vistas of northern German countryside in the times of Beethoven, Schubert, and Goethe into romantic icons, painting them at all times of night and day, around Dresden and the River Elbe, especially in the moonlight and sunlight or covered with mist. “Close your bodily eye so that you may see your picture first with the spiritual eye. Then bring to the light of day that which you have seen in the darkness so that it may react upon others from the outside inwards,” he wrote in his notes on aesthetics in 1830. Therefore, his winter landscapes were not about life in the winter but about winter itself, stark, still, desolate, where “no man has yet set his foot.”

Despite early fame and a prolific career, Friedrich lost ground in his mature years and fell into poverty, becoming the “most solitary of the solitary.” Bare trees and stumps populated with ravens and owls near graveyards and ruins filled his works, expressing the passage of time and his own state of mind. But these late paintings also explored a mystical approach, one abandoning the self to reach an intuitive understanding of physical phenomena. This period’s frisson of the sublime was later adopted by Hollywood directors to show horror, trepidation, and other emotions caused by human inadequacy against the overpowering forces of nature.

*The Polar Sea,* on this month’s cover, expresses Friedrich’s mature vision, which, far ahead of his times, was not well received. The painting was inspired by William Parry’s arctic expedition of 1819–20, a venture filled with opportunities for symbolic interpretation. The artist seized these to build a monument to nature’s triumph over human efforts to conquer it. The tiny image of the ship, inscribed HMS Griber, against a mount of ice, signals the insignificance of human enterprise. Frightful shards jut into the steel gray sky atop solid slabs of ice that form a frigid grave over what human presence might have existed before the wreck and builds a wall between the viewer and the ship.

Another leading romantic, Samuel Taylor Coleridge wrote prolifically about imagery deep with symbolism. In “The Rime of the Ancient Mariner,” he offered his version of beautiful and ominous nature, set in a metaphysical world. Among the many influences on this poem, were vivid accounts by arctic explorers. Like Friedrich, Coleridge was fascinated by their travails, which he immortalized. Here is the Mariner’s ship in the grip of polar ice: “And now there came both mist and snow, / And it grew wondrous cold: / The ice, mast high, came floating by / As green as emerald…. / The ice, was here, the ice was there, / the ice was all around: / It cracked and growled, and roared and howled, / Like noises in a swound!/”

The “rime” in the world of both Friedrich and Coleridge is symbolic of the sublime world of nature. At once fascinating and terrifying, it changes forms: water, ice, mist―taxing visual awareness, toying with the artist, challenging the scientist, tempting the poet. When European mariners were searching for the Northwest Passage, formidable polar ice lay between them and navigation. The routing was lined with myth and uncertainty, hunger, and scurvy. How times have changed! Now instead of the powerful solidity of ice, we fear instead its fragility as the polar ice cap threatens to melt into the sea, exposing among other puzzles, the dynamic evolutionary interface between human viruses and the ice that can preserve and protect them for thousands of years. What remains constant is nature’s upper hand.

In 1918, as explorers were plowing their way into the Arctic, other events were also making history. World War I was coming to a close, yet weary humanity already had a new serious concern, one that was to cause more deaths around the globe than this and future wars combined. The public health emergency spread widely in the fall of the year. Only 3 days after taking sail, the *Forsete* arrived at Longyearbyen, a tiny village in Spitsbergen Island, Svalbard, Norway, north of the Arctic Circle. An outbreak of flu had broken out on the ship caused as it turned out by an extraordinarily potent strain that would become known as Spanish Flu. Many passengers, young miners, were hospitalized and over the next few weeks, seven of them died. Their bodies, containing the deadliest flu virus the world has ever known, were buried in the local cemetery, 800 miles from the North Pole.

Almost 8 decades later, a similar grave in Alaska permafrost held valuable clues about the Spanish Flu pandemic. Unlike the one concocted in Friedrich’s imagination, this grave was not a monument to human failure. Its contents enabled RNA sequencing of much of the 1918 virus. As the ice melts, more secrets of the great pandemic may see the light of day, guiding present flu prevention activities. Moreover, other illnesses become endemic in new areas as a result of changes in climate. Tick-borne encephalitis seems to be moving northwards in Europe and shifting upwards on 84 mountains apparently influenced by such changes. Frozen solid or melting fast, sublime nature rules.
